# A Case Report of Nodular Regenerative Hyperplasia and Non-cirrhotic Portal Hypertension Post Oxaliplatin Chemotherapy

**DOI:** 10.7759/cureus.28740

**Published:** 2022-09-03

**Authors:** Nagapratap Ganta, Ankita Prasad, Mina Aknouk, Kajal Ghodasara, Ambica Nair, Zehra Taqvi, Pramil Cheriyath

**Affiliations:** 1 Internal Medicine, Hackensack Meridian Health Ocean University Medical Center, Brick, USA; 2 Research, Hackensack Meridian Health Ocean University Medical Center, Brick, USA

**Keywords:** lynch syndrome, colon carcinoma, oxalipatin, sclerotherapy, variceal banding, banding, variceal bleeding, nodular regenerative hyperplasia, non cirrhotic portal hypertension

## Abstract

Oxaliplatin is widely used in chemotherapeutic regimens for colorectal carcinoma, its recurrence, and metastasis, and is associated with better outcomes. However, oxaliplatin use is associated with injury to hepatic sinusoidal endothelium and the development of nodular regenerative hyperplasia (NRH) in the liver, which can be differentiated from nodular hyperplasia of cirrhosis by the presence of diffuse micronodular transformation without a fibrous band and the lack of perinuclear collagen tissue. This causes non-cirrhotic portal hypertension (NCPH), which presents with splenomegaly and variceal bleeding and preserved synthetic liver function. Its treatment revolves around managing variceal bleeding with banding, sclerotherapy, and beta blockers. Some patients may end up requiring liver transplantation because of recurrent variceal bleeding. We present the case of a 46 years old female who presented with recurrent variceal bleeding due to NCPH and NRH six years after treatment of colon carcinoma with oxaliplatin.

## Introduction

Portal hypertension (PHT) is defined as portal venous pressure greater than 5 mm Hg and is considered clinically significant if more than 10 mm Hg [1]. It is due to increased resistance to blood flow in portal veins formed by splenic and superior mesenteric veins. PHT is classified as hepatic, prehepatic, and post-hepatic. If resistance to flow is within the liver, it's further classified into sinusoidal, pre-sinusoidal, or post-sinusoidal, depending on where the hepatic origins of resistance lie. Prehepatic PHT is due to portal vein thrombosis, and posthepatic PHT occurs in constrictive pericarditis or Budd-Chiari syndrome. PHT typically manifests as gastrointestinal (GI) bleeding due to the development of esophageal and gastric varices as a part of collateral circulation to circumvent the resistance. It also causes splenomegaly, venous collaterals, and liver failure symptoms such as ascites and encephalopathy. Non-cirrhotic portal hypertension (NCPH) occurs due to intrahepatic or pre-hepatic lesions without cirrhosis.

Oxaliplatin is an alkylating antineoplastic agent used in combination with chemotherapy regimens to treat colorectal cancer and its metastasis or recurrence. Oxaliplatin-based combinations have significantly improved colorectal cancer patient outcomes [2]. It is associated with sinusoidal endothelial damage in the liver and causes nodular regenerative hyperplasia (NRH) [3], which causes NCPH [4], manifesting as GI bleeding (GIB). NCPH is also caused by other autoimmune, hematological, viral, neoplastic, or drug-related causes. We report the case of a 46-year-old woman who underwent FOLFOX (folinic acid, fluorouracil, and oxaliplatin) chemotherapy for colorectal cancer and developed variceal bleeding secondary to NCPH with NRH six years later. Genetic testing revealed that she had the MLH1 gene linked to Lynch syndrome.

## Case presentation

A 46-year-old female presented to the emergency room (ER) with one episode of coffee-ground vomiting and three episodes of black tarry stools. She denied any abdominal pain, diarrhea, GIB, or the use of any anti-inflammatory and anticoagulant drugs. Her medical history was significant for colon carcinoma six years before the presentation. She had undergone a right hemicolectomy and received FOLFOX chemotherapy and has been in remission since then. She also tested positive for the MLH1 gene seen in patients with Lynch syndrome, earlier known as hereditary non-polyposis colorectal cancer (HNPCC). Other pertinent surgical history includes cholecystectomy and appendectomy. Her medical history was also significant for anemia; she received intravenous iron therapy and cyanocobalamin 1000 mg/ day. Her family history was important for colon cancer in her father; she was a former smoker and occasionally took alcohol. At the presentation, she was alert and pale, with no jaundice or stigmata of liver disease. Her heart rate was 78/minute, and her blood pressure was 116/82 mm Hg. Her Initial laboratory investigation results are given in Table1 with normal serum creatinine, liver enzymes, and cardiac enzymes.

**Table 1:** Laboratory Investigations at admission BUN: blood urea nitrogen

Test	Result	Normal values
Complete blood count	14.4 10*3/uL	4.5-11.0 10*3/uL
Red blood cells	3.66 10*6/uL	4.10-5.10 10*6/uL
Hemoglobin	9.5 g/dL	12.0-16.0 g/dL
Hematocrit	29.20%	35.0-48.0 %
Prothrombin time/ International Normalized Ratio (PT/INR)	14.9 sec /1.30	11.6 sec/0.88 - 1.15
Glucose	182 mg/dl	70-99 mg/dl
BUN	38 mg/dl	5-25 mg/dl
Sodium	135 mmol/L	136-145 mmol/L

**Figure 1:**
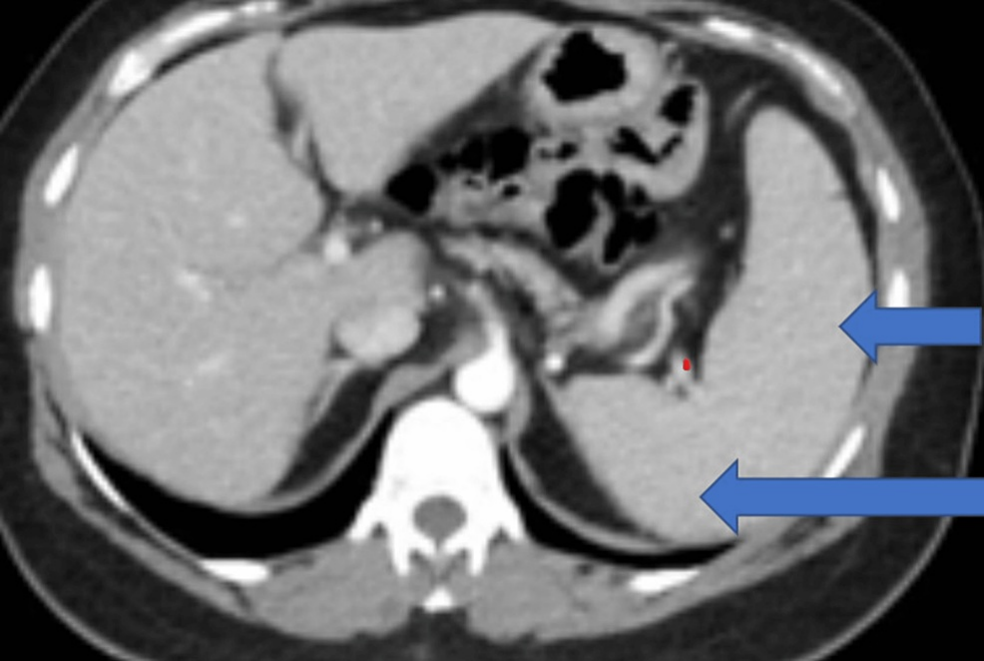
CECT abdomen showing splenomegaly (blue arrows) CECT: contrast-enhanced computed tomography

Computed tomography abdomen and pelvis with contrast showed scarring of the abdominal wall attributed to prior surgery and splenomegaly with a trace peri splenic fluid (Figure 1).

**Figure 2:**
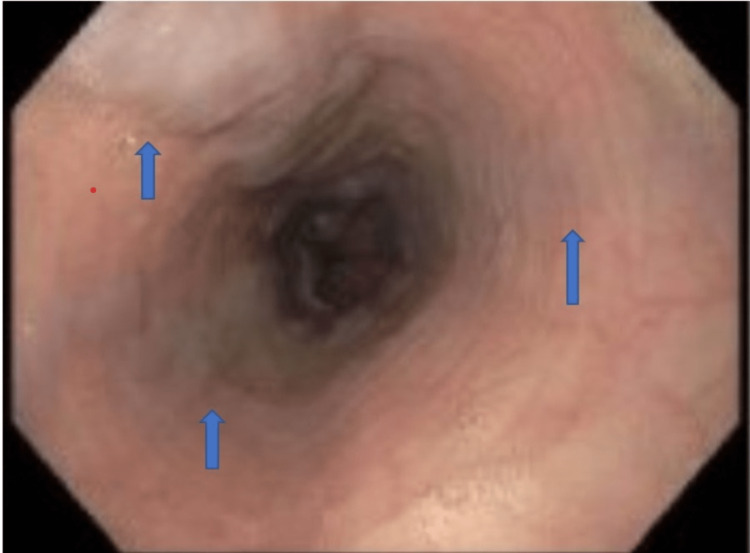
Upper GI endoscopy showing grade II varices in the lower third of esophagus (blue arrows)

**Figure 3:**
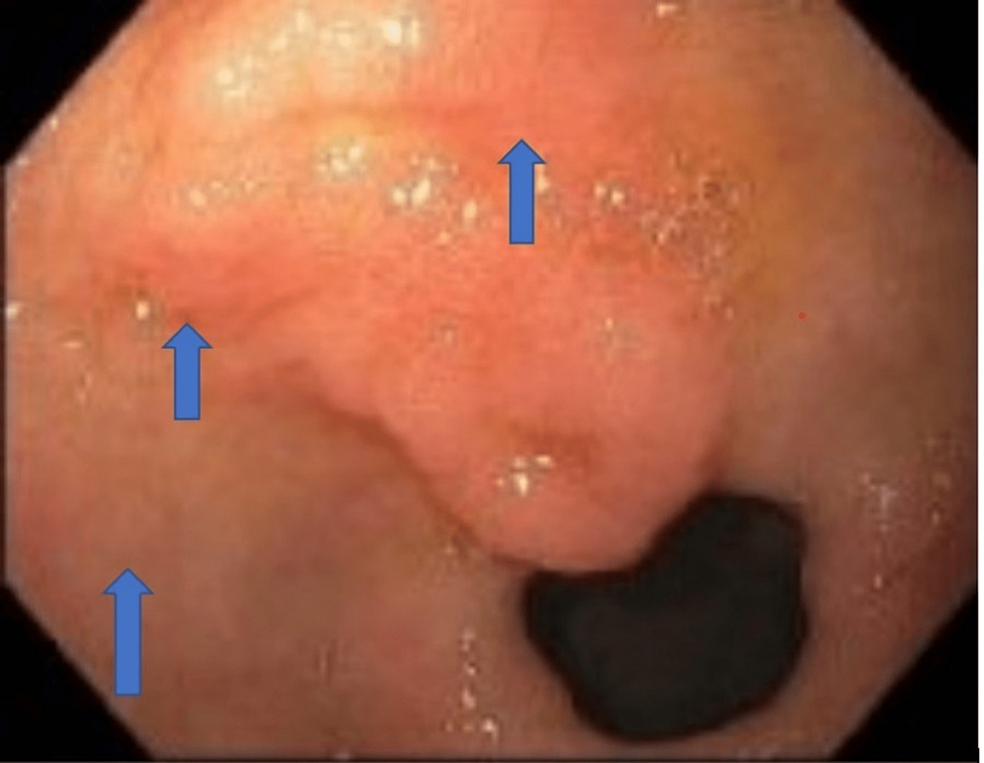
Upper GI endoscopy showing gastric antral ulcers (blue arrows)

A positron emission scan was done to rule out cancer recurrence. She had an upper GI endoscopy that showed stigmata of recent bleeding, a widened Schatzki ring in the distal esophagus, and non-bleeding gastric antral ulcers. Banding of two grade II esophageal varices was done (Figures 2, 3).

She received pantoprazole and nadolol for GIB and had a workup including a liver biopsy for possible liver disease. The histopathology reported reticulin stains highlighting parenchymal nodularity and no significant fibrosis (on reticulin and trichrome stain), suggestive of nodular regenerative hyperplasia. There was minimal steatosis and no significant portal or lobular inflammation; therein showed mild iron deposition zone macrophages and hepatocytes, but there was no evidence to support alpha one antitrypsin deficiency on Periodic Acid-Schiff with diastase (PAS-D) stain and normal glycogen stores. The histopathology was suggestive of NRH, which is known to cause NCPH. NRH can be associated with drug autoimmune diseases, myeloproliferative disorders, and vasculitis, which were ruled out in further laboratory evaluation (Table 2).

**Table 2:** Laboratory investigations

Test	Test Results	Normal Values
White blood count	14.4 10*3/uL	4.5-11.0 10*3/uL
Red blood cell count	3.66 10*6/uL	4.10-5.10 10*6/uL
Hemoglobin	9.5 g/dL	12.0-16.0 g/dL
Hematocrit	29.20%	35.0-48.0 %
Blood urea nitrogen	38 mg/dL	5-25 mg/dL
Serum sodium	135 mmol/L	136- 45 mmol/L
Alpha one antitrypsin (AAT)	180mg/dl/PiMM	100-300mg/dl
Anti-nuclear antigen (ANA)	<1:80	>1:160
Anti-smooth muscle antibody (ASMA)	<1:20	<1:20
Anti-mitochondrial antibody (AMA)	20 IU/ml	<35 IU/ml
Immunoglobulin levels (IgG, IgM, IgA)	Within normal limits	
Hepatitis A (IgM)	Negative	
Hepatitis B (HbsAg	Negative	
Hepatitis C (HCVIgM)	Negative	
Serum ferritin	12 mcg/L	40-200 mcg/L
Transferrin saturation	4%	20-50%

Finally, the presence of NRH was attributed to the use of oxaliplatin for colon cancer treatment. She was later discharged in a stable state on oral pantoprazole and nadolol and is currently being followed up by a hepatologist with six-monthly upper GI endoscopies and periodic blood tests for liver functions.

## Discussion

PHT is classified as cirrhotic PHT and NCPH, which can be further differentiated by the hepatic venous pressure (HVP), which is the difference between the wedged HVP and the free HVP [5]. Higher HVP in cirrhotic PHT is due to sinusoidal resistance [6]. In non-cirrhotic PHT, HVP is normal or only mildly elevated [6]. This patient's histopathology report confirmed NCPH due to NRH. NRH causes NCPH, which progresses slowly, remaining asymptomatic for long periods till the complications of PHT like variceal bleeding and splenomegaly develop. Variceal bleeding is a life-threatening GI emergency in PHT. A histopathological examination is required to look for a diffuse micronodular transformation of the liver without any fibrous septa to demonstrate NRH. The lack of perinuclear collagen tissue distinguishes NRH from typical regenerative nodules in cirrhotic liver disease [7]. NRH is supposedly caused by a local portal venous hypoperfusion that induces hepatocyte atrophy and apoptosis while maintaining or increasing perfusion to the surrounding acini cells. The hyperperfused areas have increased cell growth activators, which act as autocrine or paracrine peptides [8]. Another hypothesis suggests obliterative portal venopathy, which is described by the embolization of the portal venules by platelet aggregates or thrombi, causing vascular inflammation and fibrosis [8].

Sinusoidal obstruction syndrome is a complication of oxaliplatin chemotherapy, with NRH being the most common result of this injury. It causes sinusoidal dilatation and central congestion, which is attributed to damage to sinusoidal endothelial lining cells [9]. The NRH in our patient can be explained by the endothelial injury caused by oxaliplatin. NRH presents incidentally in a liver biopsy done for some other cause or as PHT [9], as our patient presented with 14 cm of splenomegaly, bleeding oesophageal varices, and normal liver enzymes. According to a study done by Huang et al., the median period from the start of chemotherapy to the diagnosis of gastroesophageal varices in oxaliplatin was 50.4 months, with a range of 7-165.4 months [10]. Our patient was diagnosed with a variceal bleed approximately 60 months after chemotherapy.

NCPH is diagnosed based on clinical, radiological, and histological findings. Liver biopsy documents the absence of cirrhosis and helps establish the diagnosis by identifying specific histological features such as NRH. Variceal bleeding is the most common presentation seen in 85-95% of patients with NCPH [8]. Recurrent variceal bleeding can be prevented by variceal ligation, sclerotherapy, and beta-blockers. Transjugular intrahepatic portosystemic shunt (TIPS) is also effective in patients with recurrent variceal bleeds. Patients in whom the variceal bleeding is controlled have an excellent overall prognosis and survival. Some patients may require liver transplantation because of recurrent uncontrolled variceal bleeding.

## Conclusions

NRH following oxaliplatin therapy is explained by injury to the sinusoidal endothelial lining in hepatic tissue, which causes sinusoidal obstruction and subsequent formation of regenerative nodules. It may remain asymptomatic or present as variceal bleeding. However, bleeding in NCPH can be readily controlled and prevented by variceal ligation, sclerotherapy, and beta-blockers. NCPH due to NRH should be considered in patients with splenomegaly or other signs of PHT in patients who have undergone treatment with oxaliplatin for colorectal carcinoma and are in remission.

## References

[1] Oliver TI, Sharma B, John S (2022). Portal hypertension. StatPearls [Internet].

[2] Cassidy J, Hochster H (2003). New oxaliplatin-based combinations in the treatment of colorectal cancer. Colorectal Dis.

[3] van den Broek MA, Olde Damink SW, Driessen A, Dejong CH, Bemelmans MH (2009). Nodular regenerative hyperplasia secondary to neoadjuvant chemotherapy for colorectal liver metastases. Case Rep Med.

[4] Kleiner DE (2015). Noncirrhotic portal hypertension: pathology and nomenclature. Clin Liver Dis (Hoboken).

[5] Bochnakova T (2021). Hepatic venous pressure gradient. Clin Liver Dis (Hoboken).

[6] Schouten JN, Verheij J, Seijo S (2015). Idiopathic non-cirrhotic portal hypertension: a review. Orphanet J Rare Dis.

[7] Hartleb M, Gutkowski K, Milkiewicz P (2011). Nodular regenerative hyperplasia: evolving concepts on underdiagnosed cause of portal hypertension. World J Gastroenterol.

[8] Sarin SK, Kumar A, Chawla YK (2007). Noncirrhotic portal fibrosis/idiopathic portal hypertension: APASL recommendations for diagnosis and treatment. Hepatol Int.

[9] Seo AN, Kim H (2014). Sinusoidal obstruction syndrome after oxaliplatin-based chemotherapy. Clin Mol Hepatol.

[10] Huang X, Li F, Wang L (2020). Endoscopic treatment of gastroesophageal variceal bleeding after oxaliplatin-based chemotherapy in patients with colorectal cancer. Endoscopy.

